# Supplementary material for: Phospholipids of APOE lipoproteins activate microglia in an isoform-specific manner in preclinical models of Alzheimer’s disease
Source: Nat Commun. 2021 Jun 7;12:3416. doi: 10.1038/s41467-021-23762-0 (PMC8184801; doi:10.1038/s41467-021-23762-0)
Supplement: Supplementary file 1 — Supplementary Information [file 41467_2021_23762_MOESM1_ESM.pdf]

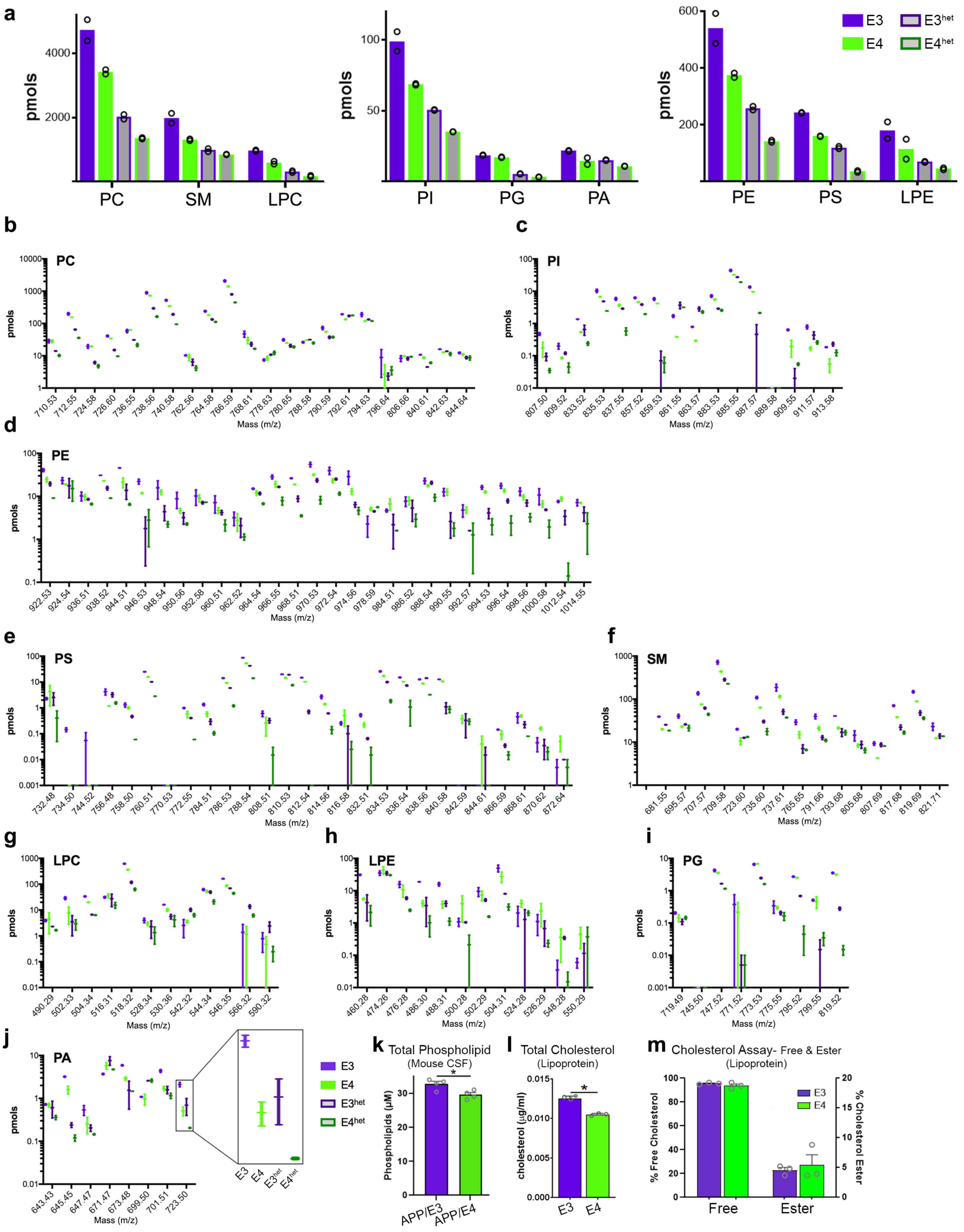
a
pmols
PC
SM
LPC
PI
PG
PA
PE
PS
LPE
E3
E4
E3het
E4het
b
PC
pmols
710.53 712.55 724.58 726.60 736.55 738.56 740.58 762.56 764.58 766.59 768.61 778.63 780.65 788.58 790.59 792.61 794.63 796.64 806.66 840.61 842.63 844.64
Mass (m/z)
c
PI
807.50 809.52 833.52 835.53 837.55 857.52 859.53 861.55 863.57 883.53 885.55 887.57 889.58 909.55 911.57 913.58
Mass (m/z)
d
PE
922.53 924.54 936.51 938.52 944.51 946.53 948.54 950.56 952.58 960.51 962.52 964.54 966.55 968.51 970.53 972.54 974.56 978.59 984.51 986.52 988.54 990.55 992.57 994.53 996.54 998.56 1000.58 1012.54 1014.55
Mass (m/z)
e
PS
732.48 734.50 744.52 756.48 758.50 760.51 770.53 772.55 784.51 786.53 788.54 808.51 810.53 812.54 814.56 816.58 832.51 834.53 836.54 838.56 840.58 842.59 844.61 866.59 868.61 870.62 872.64
Mass (m/z)
f
SM
681.55 695.57 707.57 709.58 723.60 735.60 737.61 765.65 791.66 793.68 805.68 807.69 817.68 819.69 821.71
Mass (m/z)
g
LPC
490.29 502.33 504.34 516.31 518.32 528.34 530.36 542.32 544.34 546.35 566.32 590.32
Mass (m/z)
h
LPE
460.28 474.26 476.28 486.30 488.31 500.28 502.29 504.31 524.28 526.29 548.28 550.29
Mass (m/z)
i
PG
719.49 745.50 747.52 771.52 773.53 775.55 795.52 799.55 819.52
Mass (m/z)
j
PA
643.43 645.45 647.47 671.47 673.48 699.50 701.51 723.50
Mass (m/z)
E3 E4 E3het E4het
k
Total Phospholipid
(Mouse CSF)
Phospholipids (μM)
APP/E3
APP/E4
l
Total Cholesterol
(Lipoprotein)
cholesterol (μg/ml)
E3
E4
m
Cholesterol Assay- Free & Ester
(Lipoprotein)
% Free Cholesterol
% Cholesterol Ester
Free
Ester

Supplementary Figure 1: APOE4 native lipoproteins are less lipidated with unique lipid species profile.

(a) E3 or E4 native lipoproteins were derived from pooled samples of astrocyte conditioned media from APOE3 (purple) or APOE4 (green) primary astrocytes as well as lipoproteins derived from APOE3/Abca1^{het} (purple outline) and APOE4/Abca1^{het} (green outline) astrocytes and analyzed by shotgun lipidomics. Lipoproteins derived from E3^{het} and E4^{het} astrocytes were used as negative controls and demonstrated half of the phospholipid amount of their wild-type counterparts. Note the reduction of phospholipid level in the following order: E3 > E4 > E3^{het} > E4^{het}. Lipid species-specific analysis of Phosphatidylcholine, PC (b); Phosphatidylinositol, PI (c); Phosphatidylethanolamine, PE (d); Phosphatidylserine, PS (e); Sphingomyelin, SM (f); Lysophosphatidylcholine, LPC (g); Lysophosphatidylethanolamine, LPE (h); Phosphatidylglycerol, PG (i); and Phosphatidic acid, PA (j). (k) Total phospholipid concentration of CSF from APP/E3 (purple) and APP/E4 (green) mice (14 mo) was measured using a commercial kit (APP/E3 vs. APP/E4, p = 0.0353). n=4 per group (l) Cholesterol concentration (E3 vs. E4, p = 0.014) and (m) percent free cholesterol and cholesterol ester of the native E3 (purple) and E4 (green) was measured using a commercial kit. Bars represent mean ± SEM and Box and Whiskers represent the interquartile range and mean. * $p<0.05$; determined by two-tailed unpaired t test.

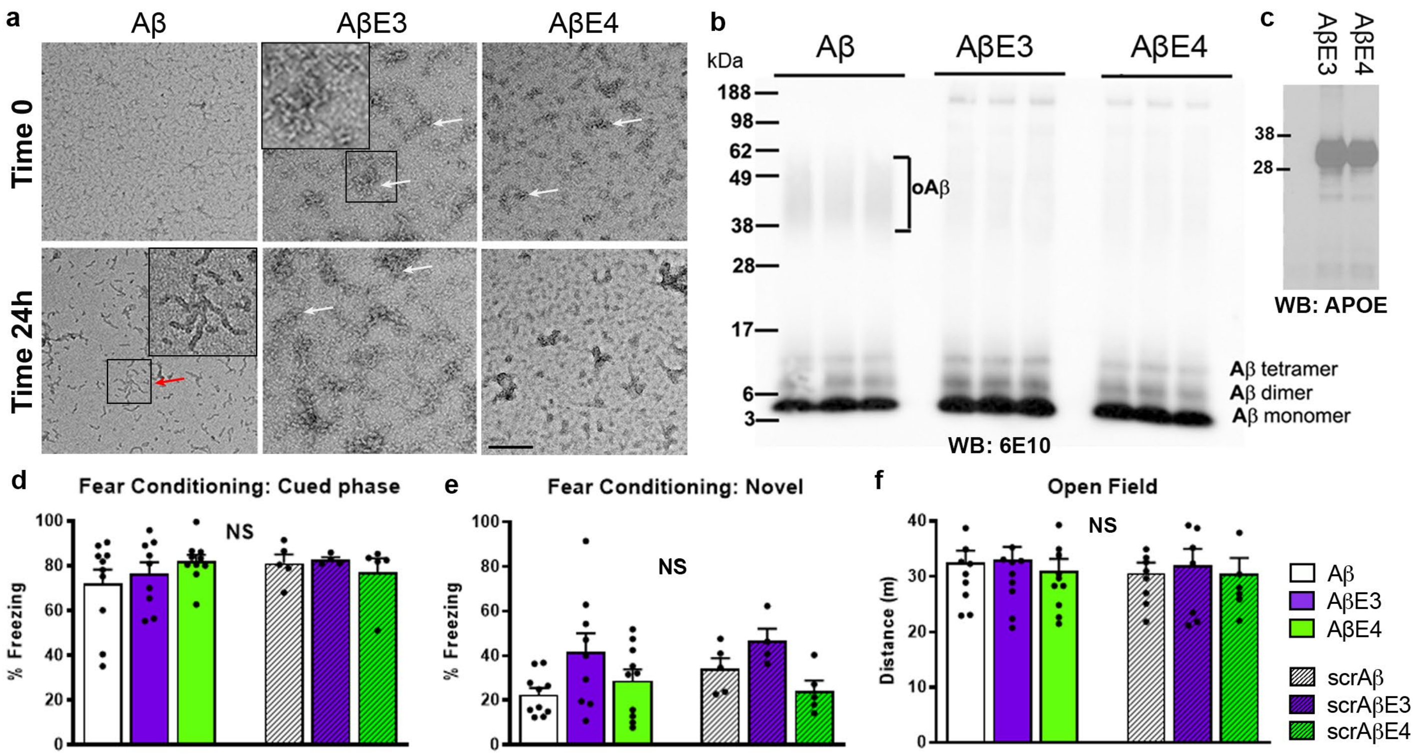

Supplementary Figure 2: Effect of native E3 and E4 lipoproteins on Aβ aggregation and behavior.

(a-c) The effect native E3 and E4 lipoproteins on Aβ aggregation was tested using a disaggregated Aβ monomer as described in the methods. Monomeric Aβ42 (10 μM) was pre-incubated with either PBS or native E3 or E4 lipoproteins (1 μM) for 24 h at oligomer forming conditions. The presence of Aβ oligomers after 24 h incubation was determined by electron microscopy (a) and Western blotting (WB) (b). (a) Representative electron micrographs of Aβ +/- APOE at the start of incubation (Time 0) and after 24 h (Time 24 h). Red arrow and inset point to Aβ oligomers in Aβ + PBS preparations. White arrows and inset point to native APOE lipoproteins that are visualized as discs. Scale bar is 100 nm. n=4 independent preparations. (b) Aliquots from the same Aβ42 preparations were resolved on SDS-NuPAGE gel and probed with anti-Aβ antibody (6E10). Brackets point toward Aβ oligomers. (c) WB for APOE demonstrates the same amount of E3 and E4 was used. (d-f). Behavior controls show no significant differences between the groups for fear conditioning and novel object recognition tests. Aβ (white), AβE3 (purple) and AβE4 (green) were infused into the cortex of WT mice and cognition examined. Scrambled Aβ (scrAβ) was used as negative controls (scrAβ, scrAβE3, and scrAβE4, line pattern). All mice were infused 30 min prior to training on days 2 through 6 of behavioral testing. (d-e) There is no significant difference in percent freezing during the cued phase (d) or novel phase (e) of the contextual-cued fear conditioning for all experimental groups assessed. (f) There is no significant difference in total distance (m) traveled during the open field during day 1 of the novel object recognition. Both suggest that differences in performance during fear conditioning and novel object recognition are due to changes in cognitive function. Analysis by one-way ANOVA followed by Tukeys multiple comparison test. n=12 for Aβ +/- E3 or E4, and n=8 for scrAβ controls. Bars represent mean ± SEM. NS no significance.

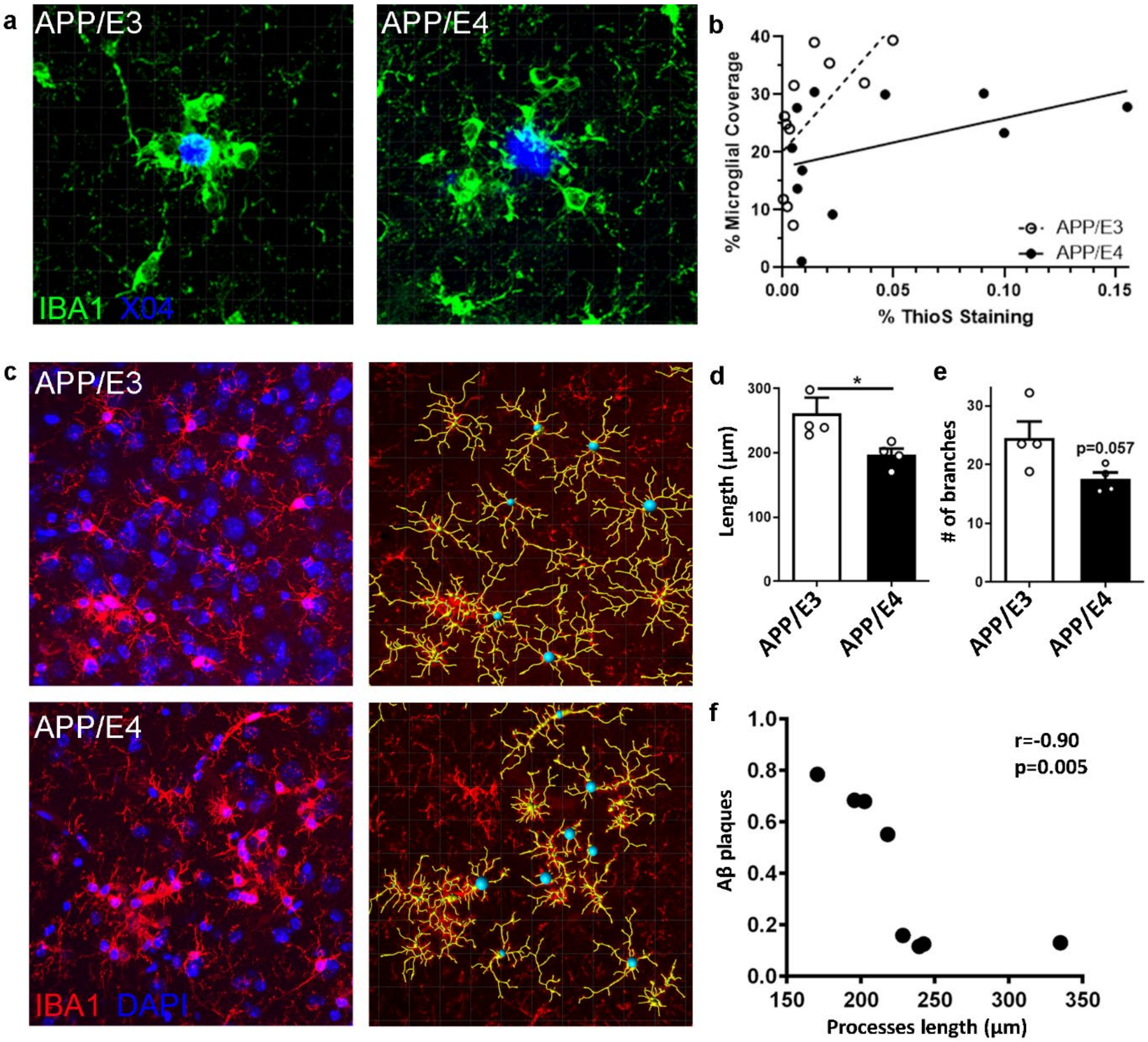

Supplementary Figure 3: Microglial barrier surrounding amyloid plaques and microglia morphology depend on APOE isoform

APP/E3 and APP/E4 mice at age of 4-6 months were used. (a-b) Mice were injected with *in vivo* amyloid labeling dye X04 and sacrificed one month later. Plaque-associated microglia barrier was assessed around X04-positive plaques using postmortem staining with IBA1. Plaque growth one month after X04 injection was evaluated by assessing the total area of ThioS staining post-mortem as described in the methods. (a) Representative confocal images of an amyloid plaque (X04 in blue) with IBA1 (green). (b) Linear regression analysis demonstrates a strong relationship between microglia barrier and Aβ plaques in the cortex of APP/E3 mice (n=11; $r = 0.64$, $p<0.01$, white dots) but not in APP/E4 mice (n=10; $r = 0.44$, p= 0.1734, black dots). (c) Representative confocal images of DAPI (blue) with IBA1 (red) with Imaris tracings of microglial processes in yellow. (d) Microglia process length is significantly decreased ($p = 0.0286$) and (e) the number of branches shows a trend towards decrease in APP/E4 (black) mice compared to APP/E3 (white); which confirms the results of our infusion model. Bars represent mean ± SEM. * $p<0.05$. Analysis was performed by two-tailed Mann–Whitney test. n=4 per group (f) Nonparametric correlation analysis demonstrates a negative correlation between microglia process length and Aβ plaques in the cortex. Spearman coefficient $r = -0.9$, $p<0.01$.

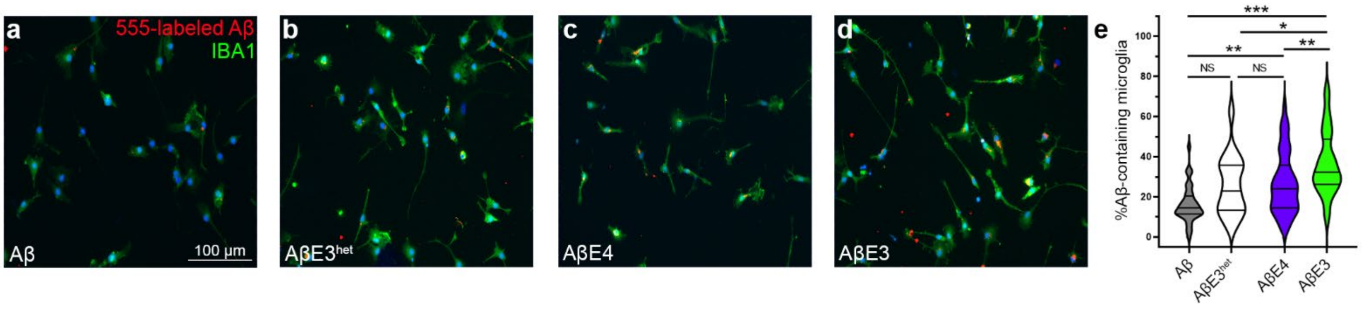

Supplementary Figure 4. Aβ uptake is differentially affected by native APOE lipoproteins *in vitro*. 555-labeled Aβ was pre-incubated with APOE native particles or PBS as indicated in the methods. Primary microglial cultures established from WT pups were treated for 1 h with Aβ+PBS (Aβ, gray), AβE3 (purple) or AβE4 (green) followed by IBA1 and DAPI immunostaining. Aβ pre-incubated with lipoproteins isolated from E3/Abca1^{het} astrocytes (AβE3^{het}, white) was used as negative control. Representative images from microglia incubated with Aβ only (a), AβE3^{het} (b), AβE4 (c) or AβE3 (d) with IBA1 in green, 555-labeled Aβ in red, and DAPI in blue. Scale bar=100 µm. (e) Bar plot showing the percent of microglia (IBA1) which co-localize to Aβ signal (555-label). Analysis by one-way ANOVA followed by Tukey's multiple comparison test. Aβ vs. AβE4, p = 0.0026; Aβ vs. AβE3, p < 0.0001; AβE4 vs. AβE3, p = 0.0019; AβE3^{het} vs. AβE3, p = 0.0206. n=3 independent cultures in triplicate per experimental group. Violin plots represent kernel densities for each dataset showing median (middle line) and 75% (top) and 25% percentile (bottom). * p<0.05; ** p<0.01; *** p<0.001; NS not significant.

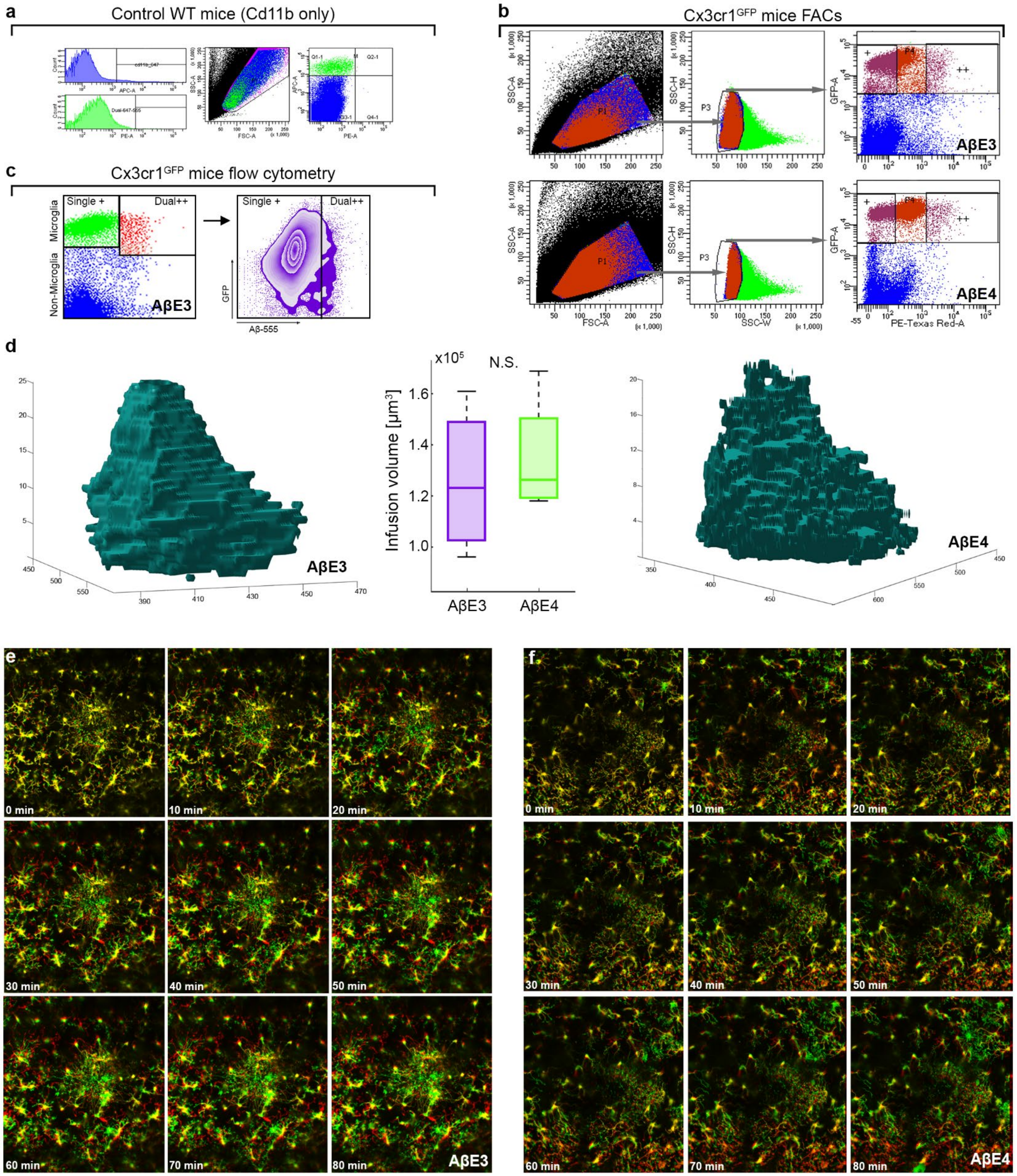

a
Control WT mice (Cd11b only)
b
Cx3cr1GFP mice FACs
c
Cx3cr1GFP mice flow cytometry
Single +
Dual++
Microglia
Non-Microglia
AβE3
AβE4
GFP
Aβ-555
SSC-A
FSC-A
SSC-H
SSC-W
GFP-A
PE-Texas Red-A
P1
P3
P4
d
Infusion volume [µm3]
N.S.
e
f

Supplementary Figure 5: *In vivo* microglial interaction with Aβ is affected by APOE isoform over time.

Cx3cr1^{GFP} mice were infused with control, or 555-labeled Aβ42 pre-incubated with E3 (AβE3) or E4 (AβE4). a-c: Flow cytometry and FACS. Q1-1 corresponds to cells that are CD11b^{High}/Aβ-555^{Low} and are counted as single+ cells. The Q2-1 correspond to CD11b^{High}/Aβ-555^{High} cells counted as dual+. (a) WT control injected mice show no cells in Q2-1 quadrant labeled as dual positive. (b) FACS results showing all cells, followed by removing multiple cells, and finally separating by microglia marker high (Cx3cr1^{GFP}) and 555-labeled Aβ. This allows for the identification of single+ and dual+ cells in Cx3cr1^{GFP} mice. FACS gating used for Figure 4. (c) Gating for flow cytometry and zebra plots generated from all gated microglia showing both single+ and dual+ populations in Figure 3b-c. (d-f), Two-photon imaging of Hilyte™-555-labeled Aβ pre-incubated with native E3 or E4 particles and microglia tracked in real time. (d) We injected the same infusion volume for all animals and post-injection we acquired *in vivo* three-dimensional z-stacks to verify the biodistribution. Z-stacks to verify the biodistribution had two color channels - red channel (595/50 nm) to capture the spread of the AβE3 (purple) or AβE4 (green) infusion and green channel (525/50 nm) to establish the initial position of the microglia within the injected volume. The in-plane matrix size was 1024x1024 pixels with an effective resolution of 0.8 microns/pixel. We used a step size of 5 microns along the z direction across the cortical thickness, delivering an effective tissue volume of 3.251 cubic microns per pixel. Each z-tack was collected across the full depth of the infusion site with an average stack size of 30 slices. We segmented, rendered and computed the three-dimensional volumes of each Aβ infusion site and determine that there was no difference in the rendered volumes of Aβ for AβE3 and AβE4 groups. Shown are representative infusion volume renderings from each group and Box and Whiskers. N.S. not significant by two-tailed unpaired *t* test. n=4 mice/group. The boxes represent the range from the first to third quartiles with the center line representing the median. The whiskers represent the minima and maxima. (e-f) Representative time series (n=4 mice/group) are color coded with the initial time point in red and later time points in green as indicated below each image from 0-80 min post injection from AβE3 (e) or AβE4 (f) groups.

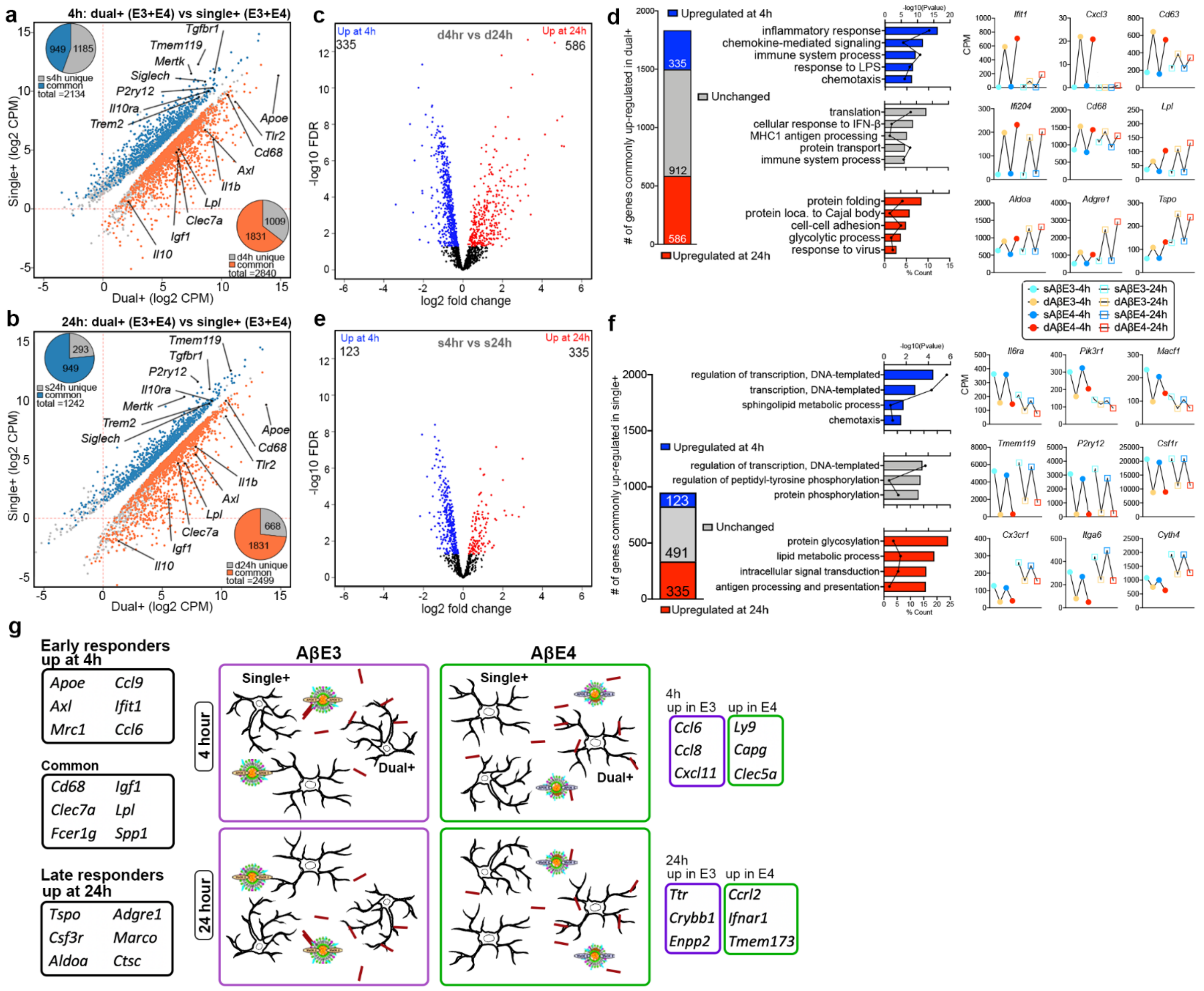

Supplementary Figure 6. Comparisons of microglial transcriptome from Cx3cr1GFP mice at 4 and 24 hours post-injection.

(a-b) Scatterplots represent significantly affected transcripts (FDR<0.05) of dual+ vs single+ microglia from Cx3cr1^{GFP} mice at the 4 h (a) or 24 h (b) timepoint. X and Y axes correspond to the log2 average of the count per million (CPM) for dual+ and single+ microglia respectively. Pie charts in the upper left (single+, blue), and lower right (dual+, orange) represent the genes common between, or unique (gray) to a single timepoint. (c-f) Comparison of common DEGs in 4 vs 24 h post-injection. Volcano plots and bar plots depict the 1831 commonly differentially expressed in dual+ cells (c; blue, up at 4h, and red up at 24 h) and 949 commonly differentially expressed in single+ cells (e; blue, up at 4h, and red up at 24 h). Grey box indicates non-significantly affected genes; blue, a higher expression at 4h, and red with a higher expression at 24 h (d and f). For each group the GO term bar plots indicate the -log10P value for each term, and the associated center point of each bar represents the percent of submitted genes found in each GO term. Line-patterning graphs show examples of genes from each category. sAβE3 light blue, dAβE3 beige, sAβE4 blue, dAβE4 red; 4hr solid circles, 24 hr open circles. n=4 mice/group. (g) Summary of early, common and late responding genes associated with different timepoints (4 or 24 h) following the Aβ infusion (left) and early and late responding genes that are uniquely affected by APOE isoform (right).

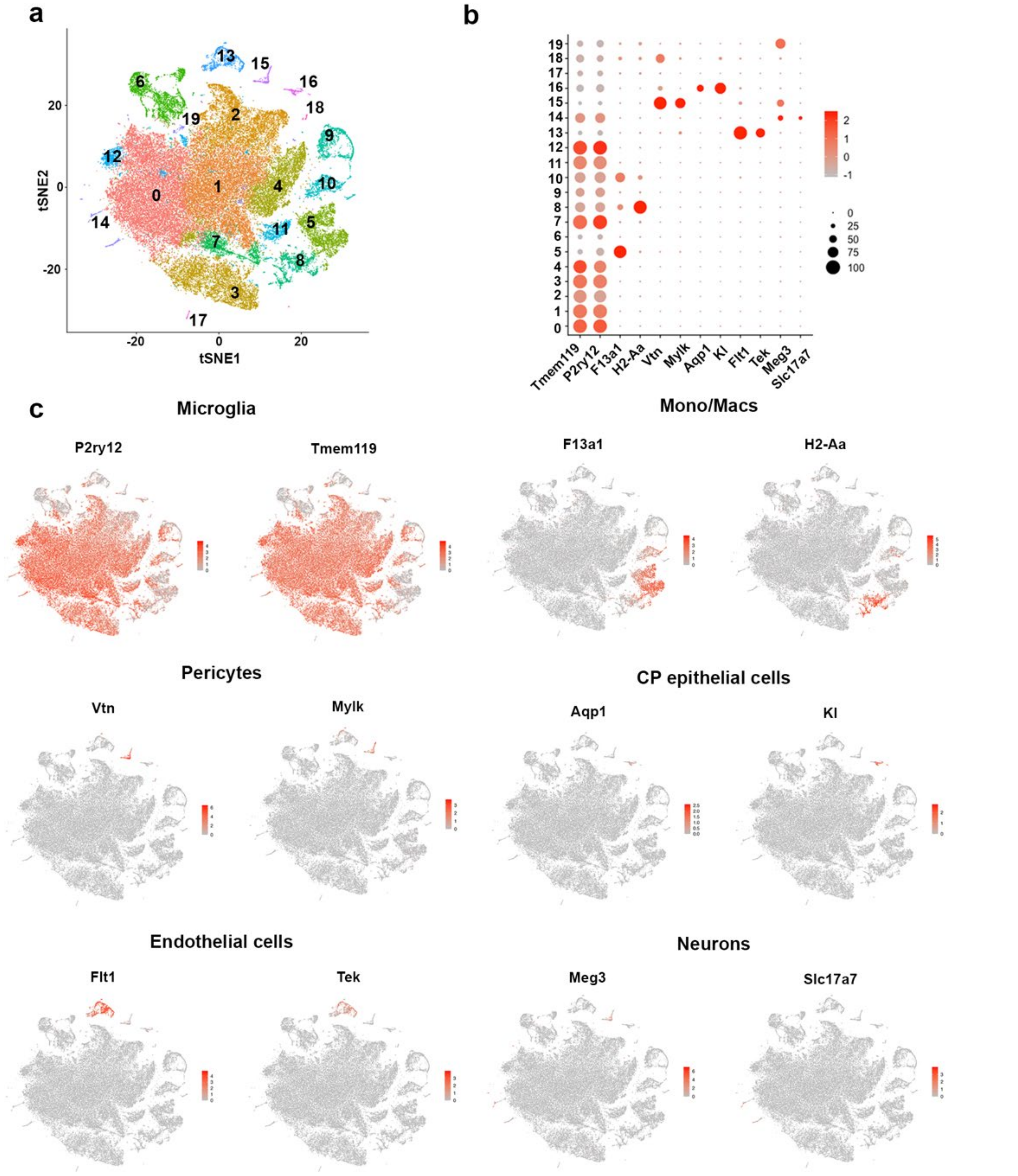

Supplementary Figure 7. scRNA-seq analysis of injected mice identifies distinct multiple cell types.

(a) t-SNE plot showing clusters of 60,046 sequenced cells based on transcriptomic similarities using Seurat. (b) Dot plot showing expression of established cell-type specific genes. (c) Feature plots showing expression of established cell specific genes. n=9 (Control, WT-AβE3, WT-AβE4, Trem2^{ko}-AβE3 and Trem2^{ko}-AβE4).

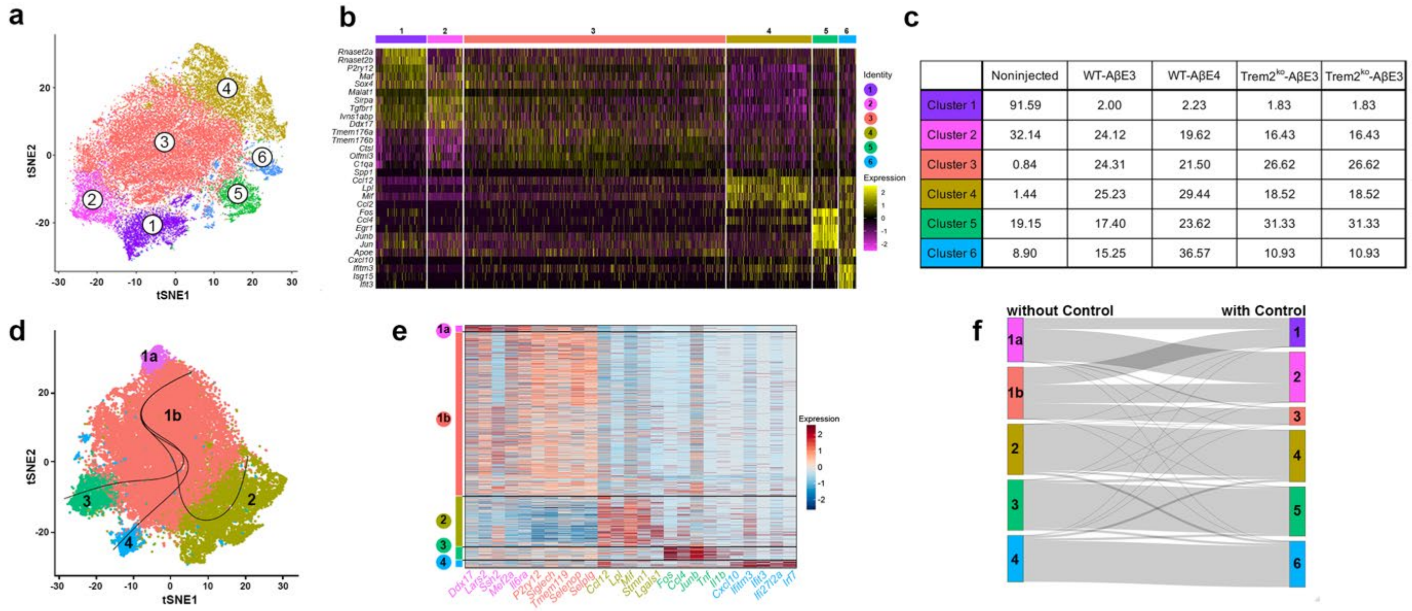

	Noninjected	WT-AβE3	WT-AβE4	Trem2^{ko}-AβE3	Trem2^{ko}-AβE3
Cluster 1	91.59	2.00	2.23	1.83	1.83
Cluster 2	32.14	24.12	19.62	16.43	16.43
Cluster 3	0.84	24.31	21.50	26.62	26.62
Cluster 4	1.44	25.23	29.44	18.52	18.52
Cluster 5	19.15	17.40	23.62	31.33	31.33
Cluster 6	8.90	15.25	36.57	10.93	10.93

Supplementary Figure 8. scRNA-seq analysis of injected and non-injected mice identifies distinct microglia subclusters.

(a) t-SNE plot showing clusters of 39,898 microglia cells after removal of contaminating cell types from non-injected and injected groups. (b) Heatmap showing the expression of top 5 upregulated genes in each microglia cluster of the same mice. n=9 (c) Table showing the proportion of cells belonging to each cluster by non-injected and injected groups. (d) t-SNE plot showing clusters of 36,244 microglial cells after re-clustering of injected samples and inferred trajectories by Slingshot. (e) Heatmap showing the expression of top 5 genes in each cluster after re-clustering of injected mice. n=8 (f) Sankey plot showing preserved clusters between the two data sets. The top 100 marker genes for each cluster were used for generation of the Sankey plot.

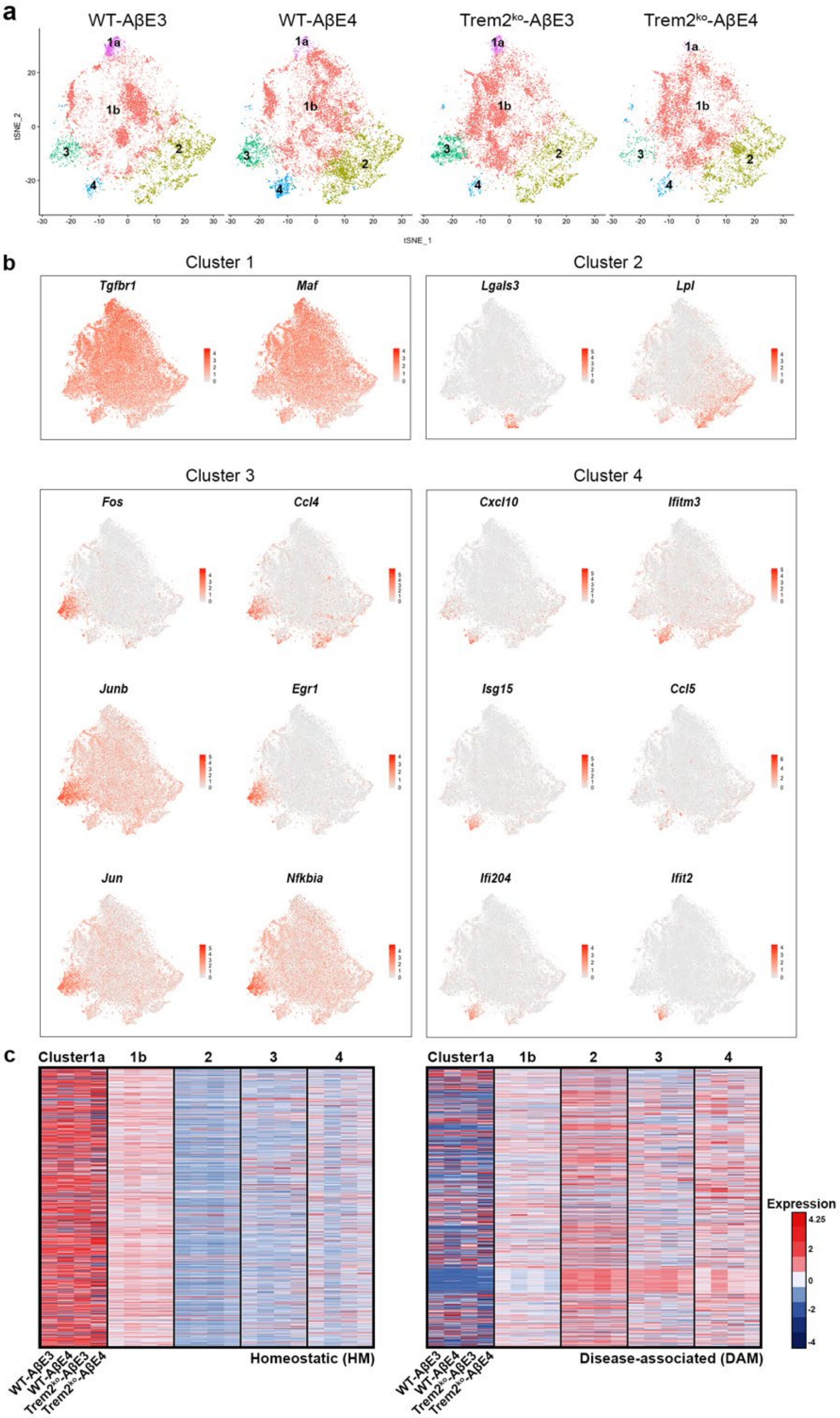

Supplementary Figure 9. scRNA-seq Microglia specific sub-clustering.

(a) t-SNE plots of microglia from each experimental condition. (b) Feature plots of representative genes in each cluster. (c) Heatmaps of homeostatic and disease-associated microglia gene expression in each cluster. n=2 per group.

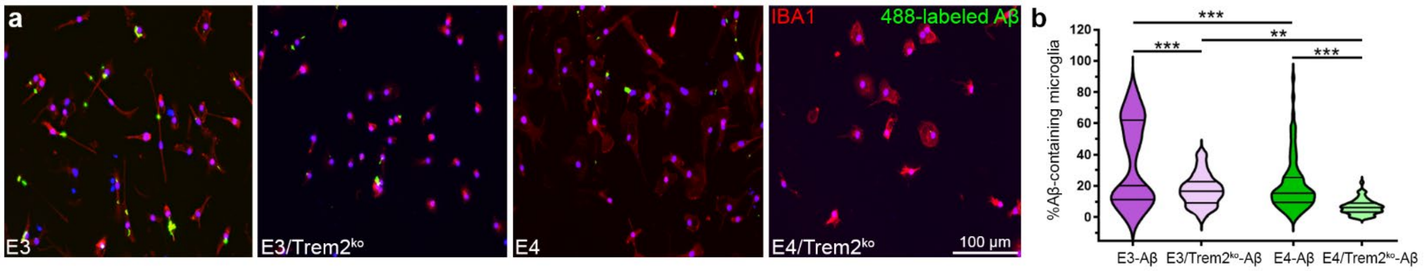

Supplementary Figure 10. *Trem2* deficiency and APOE isoform expression affect microglia response to Aβ.

Microglia established from E3, E3/Trem2^{ko}, E4, and E4/Trem2^{ko} mice were treated with and 488-labeled Aβ for 1 hour. (a) Representative images of microglia from E3, E3/Trem2^{ko}, E4, and E4/Trem2^{ko} mice labeled with IBA1 in red and 488-labeled Aβ in green from three independent cultures in triplicate for each genotype. (b) Bar plot showing the average percentage of Aβ-containing microglia for each genotype (E3 purple, E4 green, E3/Trem2^{ko} light purple, E4/Trem2^{ko} light green). Analysis by one-way ANOVA followed by Tukey's multiple comparison test. E3-Aβ vs. E3/Trem2^{ko}-Aβ, $p < 0.0001$; E3-Aβ vs. E4-Aβ, $p < 0.0001$; E3/Trem2^{ko}-Aβ vs. E4/Trem2^{ko}-Aβ, $p = 0.0024$; E4-Aβ vs. E4/Trem2^{ko}-Aβ, $p < 0.0001$. n=3 independent cultures in triplicate per experimental group. Violin plots represent kernel densities for each dataset showing median (middle line) and 75% (top) and 25% percentile (bottom). ** p<0.01; *** p<0.001.

Supplementary Table 1: Patient demographics for AD

APOE	Sex	PMI (hrs)	Age at Death	Braak Stage
3/3	*F*	Range 2.0 - 7.5	Range 71 - 87	6
3/3	*F*			
3/3	*F*			
3/3	*M*			
3/3	*M*			
3/3	*M*			
3/3	*M*			
average		4.0	81.57	6
standard deviation		1.8	5.41	0

APOE	Sex	PMI (hrs)	Age at Death	Braak Stage
3/4	*F*	Range 2.1 - 9.5	Range 75 - 90	6
3/4	*F*			
3/4	*F*			
3/4	*F*			
3/4	*F*			
3/4	*M*			
3/4	*M*			
3/4	*M*			
average		3.7	79.63	6
standard deviation		2.4	7.65	0

APOE	Sex	PMI (hrs)	Age at Death	Braak Stage
4/4	*F*	Range 2.2 - 7.0	Range 71 - 84	6
4/4	*F*			
4/4	*F*			
4/4	*M*			
4/4	*M*			
4/4	*M*			
4/4	*M*			
average		4.1	79.14	6
standard deviation		1.8	5.49	0

Supplementary Table 2: Primer List

Method	Gene	Species	Primer Sequences/Assay ID
Mouse genotyping	*Abca1*	Mouse	wtF: 5'– TGGGAACTCCTGCTAAAAT – 3'
			wtR: 5' – CCATGTGGTGTGTAGACA – 3'
			mutR: 5' – TGCAATCCATCTTGTTCAAT – 3'
			mutF: 5' – TTTCTCATAGGGTTGGTCA – 3'
	Apoe	Mouse	oIMR0180: 5' – GCCTAGCCGAGGGAGAGCCG – 3'
			oIMR0181: 5' – TGTGACTTGGGAGCTCTGCAGC – 3'
			oIMR0182: 5' – GCCGCCCCGACTGCATCT – 3'
	APOE3/4	Human	PGK: 5' – GCAGCCTCTGTTCCACATACACT – 3'
			Huexon4: 5' – TTGATTCTCCTGGGCCACTG – 3'
			Mouseintron2R: 5' – GCAAGAGGTGATGGTACTCG – 3'
			MusApoE2: 5' – GTATCTAAACAGACTCAACAGCCTC – 3'
	Cx3cr1	Mouse	14276: 5' –GTCTTCACGTTCGGTCTGGT– 3'
			14277: 5' –CCCAGACACTCGTTGTCCTT– 3'
			14278: 5' –CTCCCCCTGAACCTGAAAC– 3'
	PS	Human	13763: 5' – TCATGACTATCCTCCTGGTGG – 3'
			13764: 5' – CGTTATAGGTTTTAAACACTTCCCC – 3'
			PS339: 5' – GTAGGTGGAAATTCTAGCATCATCC – 3'
			PS338: 5' – CTAGGCCACAGAATTGAAAGATCT – 3'
	Trem2	Mouse	24466: 5' – AAGCAAGTGGCTGTCTCCTC– 3'
			24467: 5' –TGTGTACTCACCCTCCAGCA – 3'
Taqman assay	*P2ry12*	Mouse	Mm00446026_m1
	Tmem119	Mouse	Mm00525305_m1
