# Supplementary material for: Phospholipids of APOE lipoproteins activate microglia in an isoform-specific manner in preclinical models of Alzheimer’s disease
Source: Nat Commun. 2021 Jun 7;12:3416. doi: 10.1038/s41467-021-23762-0 (PMC8184801; doi:10.1038/s41467-021-23762-0)
Supplement: Supplementary file 3 — Description of Additional Supplementary Files [file 41467_2021_23762_MOESM3_ESM.docx]

Description of Additional Supplementary Files

Title: Supplementary Movie 1.

Description: Video showing time-lapse two-photon imaging of GFP-labeled microglia movement after AβE3 injection. Cx3cr1^GFP^ mice were infused with Hi-Lyte Fluor 555-labeled Aβ (Aβ-555) pre-incubated with native E3.

Title: Supplementary movie 2.

Description: Video showing time-lapse two-photon imaging of GFP-labeled microglia movement after AβE4 injection. Cx3cr1^GFP^ mice were infused with Hi-Lyte Fluor 555-labeled Aβ (Aβ-555) pre-incubated with native E4.
